# Dysphoric students show higher use of the observer perspective in their retrieval of positive versus negative autobiographical memories

**DOI:** 10.1080/09658211.2012.730530

**Published:** 2012-10-22

**Authors:** Sabine Nelis, Elise Debeer, Emily A. Holmes, Filip Raes

**Affiliations:** ^a^ Faculty of Psychology and Educational Sciences, University of Leuven, Belgium; ^b^ MRC Cognition and Brain Sciences Unit, Cambridge, UK

**Keywords:** Autobiographical memory, Vantage perspective, Depression, Dampening, Mental imagery

## Abstract

Autobiographical memories are retrieved as images from either a field perspective or an observer perspective. The observer perspective is thought to dull emotion. Positive affect is blunted in depressed mood. Consequently, are positive events recalled from an observer perspective in depressed mood? We investigated the relationship between memory vantage perspective and depressive symptoms in a student sample. Participants completed the Autobiographical Memory Test (AMT; Williams & Broadbent, 1986) and assessed the perspective accompanying each memory. The Beck Depression Inventory-II (BDI-II; Beck, Steer, & Brown, 1996) and the Responses to Positive Affect questionnaire (RPA; Feldman, Joormann, & Johnson, 2008) were administered. The results showed a small positive association between depressive symptoms and the use of an observer perspective for positive autobiographical memories, but not for negative memories. Furthermore, comparing a subgroup with clinically significant symptom levels (dysphoric students) with non-dysphoric individuals revealed that dysphoric students used an observer perspective more for positive memories compared with negative memories. This was not the case for non-dysphoric students. The observer perspective in dysphorics was associated with a dampening cognitive style in response to positive experiences.

Life events are not always remembered from the original visual perspective from which they were experienced. When we use our original perspective, called field perspective, we experience the event through our own eyes. In an observer perspective (“fly on the wall” perspective) we can see ourselves in the situation from the outside (Nigro & Neisser, 1983). Over the last decade, research has started to focus on vantage perspective (field vs. observer perspective) in autobiographical memory retrieval in depression. As the two perspectives imbue a memory with different emotional experiences (whereby field-perspective memories are more emotional and the observer perspective dulls emotions; e.g., McIsaac & Eich, 2002; Williams & Moulds, 2008) and cognitive appraisals (whereby, for example, the observer perspective may lend itself to a more comparative style of thinking; Holmes, Coughtrey, & Connor, 2008; Kuyken & Howell, 2006), perspective might have implications for emotional disorders, such as depression.

Depression has been associated with an increased use of an observer (vs. field) perspective when recalling past events (e.g., Kuyken & Howell, 2006; Kuyken & Moulds, 2009; Lemogne et al., 2006). Moreover, the emotional valence of memories may be critical. Positive affect is reduced in depressed mood (American Psychiatric Association, 1994) and the observer perspective is thought to dull emotion (e.g., Robinson & Swanson, 1993). Consequently, are positive events recalled from an observer perspective in depressed mood? Lemogne et al. (2006) asked a group of depressed patients and healthy controls to retrieve positive and negative events within a series of life periods and to subsequently assess each memory, including perspective. Depressed patients displayed an observer perspective more for positive events than for negative events, while this was not the case in the control group. In addition, compared with the control group, (formerly) depressed patients were more likely to recall positive memories from an observer perspective, but no significant difference in visual perspective for negative memories emerged (Bergouignan et al., 2008; Lemogne et al., 2006). Consequently, Bergouignan et al. (2008) and Lemogne et al. (2006) linked the field perspective deficit in depression mainly to positive memories. The fact that visual perspective depends on the concordance between the self in the recalled memory and how individuals view themselves at the moment of recall may offer a rationale for those results (see also Bergouignan et al., 2008; Kuyken & Howell, 2006; Lemogne et al., 2006). Libby and Eibach (2002) demonstrated that people tend to use an observer perspective when the visualised memory content conflicts with their current self-concept (indicating self-change), whereas a field perspective is used in memories compatible with their current self-concept. Depression is associated with a predominantly negative self-concept (e.g., Bargh & Tota, 1988; Beck, Rush, Shaw, & Emery, 1979). Given that the confrontation with positive past events conflicts with their negative self-concept, one would predict that depressed individuals recall positive past events especially from an observer perspective and negative events from a field perspective.

There is also an additional way to look at the relation between observer perspective and depression. Actions imagined from an observer perspective (vs. field) are interpreted in a more abstract way, stressing the broader meaning of one's action (Libby, Shaeffer, & Eibach, 2009). Additionally, Kuyken and Howell (2006) suggested that imagining oneself from a distance in an observer perspective makes it possible to evaluate the self in the image. Kuyken and Moulds (2009) found support for this idea. In their study, a self-report measure of negative self-evaluation was positively associated with the number of memories retrieved from an observer perspective. Consequently, with regard to positive memories, it might be the case that the observer perspective facilitates a negative evaluation of the actual positive memory. The first evidence for this came from an experimental study by Holmes et al. (2008) in which participants were asked to imagine a series of positive situations. Imagining the positive material from an observer perspective not only led to mood blunting but also a more negative mood. Holmes, Lang and Shah (2009) and Holmes et al. (2008) suggested that this effect could arise from a comparative/ruminative thinking style. Social psychology theories state that confrontation/“upwards” comparison with something positive (e.g., a person who is cleverer than you) leads to negative emotion, whereas a downwards comparison may lead to positive emotion (Morse & Gergen, 1970). Interestingly, depressed individuals are characterised by a dampening response style to positive experiences (see Feldman, Joormann, & Johnson, 2008; Raes, Smets, Nelis, & Schoofs, 2012). When depressed people start to ruminate on positive experiences (e.g., when they receive a compliment from their boss), they may “dampen” the associated positive affect (e.g., thinking that they do not deserve it or—in a comparative fashion—start to think about things that did not go well in the past). This creates a distance between the individual and the (positive) experience that is being retrieved. Such distancing would be aided by and even promote the use of an observer perspective.

The aim of the present study was to further explore the relationship between depressed mood and memory vantage perspective. First, we predicted that, overall, there would be a positive association between observer-perspective memories and depressive symptoms, and that volunteers who reported clinically significant depressive symptoms for the previous two weeks (dysphoric students) would be more likely to use an observer perspective for positive memories compared to negative memories and compared to non-dysphoric students (cf. Lemogne et al., 2006). Second, in order to gain insight into the use of an observer perspective, we wanted to verify the suggested association between observer perspective for positive memories and the use of a dampening response style to positive affect, predicting that both will be positively related.

## METHOD

### Participants

Participants were 440 first-year psychology students from the University of Leuven. Nine participants had missing data for all perspective ratings or for the Beck Depression Inventory-II (BDI-II; Beck, Steer, & Brown, 1996) and were excluded (*N*=431; 372 women; *M*_age_=18.37, *SD*=1.88). All participants received course credit. For further analyses (see below), a sample of students with a high (*N*=50) or a low (*N*=56) BDI-II score was selected. Written informed consent was obtained from all participants.

### Materials

#### 

##### Autobiographical Memory Test (AMT; Williams & Broadbent, 1986)

In the AMT, participants are given 1 minute to write down a personal memory in response to 10 cue words. Five positive and five negative cue words are presented in an alternating order: confidence (trust), scared, pleasurable, angry, courage, sad, at ease, bold, surprised and stupid.^1^ We used the “Minimal Instructions” version of the AMT (Debeer, Hermans, & Raes, 2009). In this Minimal Instructions version, participants are asked to retrieve memories without emphasising in the instructions that these should be specific. All memories have to refer to different events or situations and cannot refer to events from the past 7 days. After memory retrieval, participants were instructed to categorise the imagery perspective as first or third person as follows:

Research shows that memories can be experienced in two different ways of “seeing” or “experiencing”. (1) A memory can be experienced from a third person perspective. Using this perspective you take the position of a spectator or an observer: you can see yourself in the remembered scene. (2) Or you can experience a memory from a first person perspective. Using this perspective it seems like you experience the situation again through you own eyes, so you can't see yourself or your own actions.

^1^The original Dutch words were: *vertrouwen*, *bang*, *prettig*, *boos*, *moed*, *droevig*, *gerust*, *brutaal*, *verrast*, and *lomp*.


##### Beck Depression Inventory-II (BDI-II; Beck, Steer, & Brown, 1996)

The BDI-II measures severity of depressive symptoms and consists of 21 four-choice statements. Participants are asked to indicate which of the four statements best describes how they felt during the past two weeks. The total BDI-II score is computed by summing the 21 scores (ranging from 0 to 3) which offers a total score ranging from 0 to 63. We used the Dutch translation by Van der Does (2002). Cronbach's alpha in the present sample was .85.

##### Responses to Positive Affect questionnaire (RPA; Feldman et al., 2008)

The RPA measures responses to positive affect and consists of 17 items, ranging from 1 (“almost never”) to 4 (“almost always”). The scale is divided into three subscales: Dampening (e.g., “My streak of luck is going to end soon”), Self-focused positive rumination (e.g., “I am achieving everything”), and Emotion-focused positive rumination (e.g., “Think about how happy you feel”). We used the Dutch version, consisting of 16 items, for which adequate psychometric properties are reported (Raes, Daems, Feldman, Johnson, & Van Gucht, 2009). The current study focused on the 7-item Dampening subscale with a total score ranging from 7 to 28. Cronbach's alpha in the present sample was .77. The other subscales were not of interest in this research.

### Procedure

At the beginning of the academic year (October 2009), participants were invited by email to attend a mass test. Following informed consent, they completed the AMT, BDI-II, and RPA (in that order), and other questionnaires not of interest in this study.

### Analyses

The correlation (Pearson's *r*) between BDI-II scores and proportions of observer-perspective memories (propObs) was calculated for positive cue words (propObs-pos) and negative cue words (propObs-neg) separately.^2^ Additionally, we selected two subsamples from the total sample: a group with a BDI-II score of 20 or higher (high-BDI group; *N*=50), reflecting clinically significant levels of depressive symptomatology (Beck et al., 1996) and a group with the lowest BDI-II scores (percentile 10), ranging from 0 to 3 (low-BDI group; *N*=56). A repeated measures analysis of variance (ANOVA) was conducted with cue valence (positive vs. negative) as a within-subjects factor, BDI-II (low-BDI vs. high-BDI) as a between-subjects factor, and proportion of observer-perspective memories as the dependent variable.
^2^Memories were categorised using the valence of the cue word. To verify whether the valence of a cue word was congruent with the valence of a memory, a random sample of 40% of the AMTs from the low-BDI group and 41% of the AMTs from the high-BDI group were rated on valence by an independent researcher. Memories were coded as “rather positive”, “rather negative”, or “undecided”. AMTs were selected via an online randomisation system (http://randomizer.org). Four memories were unfinished and not included in the valence ratings. The percentage of emotional congruent memories for each cue word was as follows: 62.5% (confidence), 92.9% (scared), 95.1% (pleasurable), 92.5% (angry), 81.1% (courage), 90.5% (sad), 87.2% (at ease), 95% (bold), 85.4% (surprised), 84.6% (stupid). These percentages were considered as sufficient to draw conclusions about the content of the memories. A random sample of 20% of the AMTs in the low-BDI group and 21% in the high-BDI group were recoded by a second research assistant, resulting in an inter-rater agreement of .86 (Cohen's Kappa; Cohen, 1960).


Correlation between dampening of positive affect (as measured by the RPA) and propObs-pos was calculated for the whole sample, as well as for the low-BDI group and the high-BDI group separately.

## RESULTS

Descriptive statistics for the whole sample were as follows: propObs, *M*=0.34 (*SD*=0.21, range=0–1); propObs-pos, *M*=0.33 (*SD*=0.25, range=0–1); propObs-neg, *M*=0.36 (*SD*=0.26, range=0–1); BDI-II, *M*=10.50 (*SD*=7.03, range=0–41). There were no significant differences between the low-BDI and high-BDI group in terms of gender, χ^2^(1)=2.24, *p*=.14 and age, *F*(1, 104)=0.03, *p*=.87. Descriptives for each group are presented in Table 1. Overall, and in line with previous findings (e.g., Nigro & Neisser, 1983; Robinson & Swanson, 1993), more memories were recalled from a field perspective (66%) than from an observer perspective (34%), *t*(430)=15.27, *p*<.001.

**TABLE 1:** Descriptive statistics among the low-BDI group and the high-BDI group

	Low-BDI group (N=56)			High-BDI group (N=50)		
	Mean	SD	Min–max	Mean	SD	Min–max
Age (years)	18.41	2.70	17–37	18.48	1.09	17–22
Gender (% female)	82			92		
BDI-II	1.91	1.05	0–3	24.88	5.20	20–41
RPA-Dampening	10.02	2.69^a^	7–17	15.71	4.19^b^	7–26
Observer memories (prop)						
Positive	0.29	0.25	0–1	0.37	0.26	0–1
Negative	0.30	0.25	0–1	0.28	0.23	0–0.8
^a^*N*=54 (two missing data).						
^b^*N=*48 (two missing data).						
BDI-II=Beck Depression Inventory-II; RPA-Dampening=Dampening subscale of the Responses to Positive Affect questionnaire. The two groups significantly differed on RPA-Dampening, *F*(1, 78)=64.87, *p*<.001, Welch's *F*.						

### Correlations between depressive symptoms and the use of an observer perspective

There was no significant correlation between the overall proportion of observer-perspective memories and BDI-II score, *r*(431)=.04, *p*=.37. Taking valence into account, BDI-II score showed a small but significant positive correlation with propObs-pos, *r*(431)=.10, *p*=.03, but not with propObs-neg, *r*(431)=−.02, *p*=.61. These latter two correlations are significantly different, *z*=2.41, *p*=.02 (Steiger's Z-test).

### Comparing high- and low-BDI groups on visual perspective in memory retrieval

**Figure 1.**
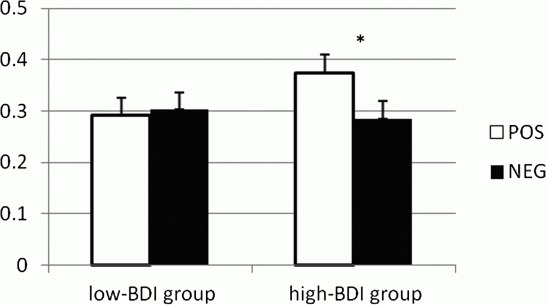
Mean proportion of observer-perspective memories (+*SEM*) for the positive and negative cue words in a low-BDI group and a high-BDI group. *p<.05.

Figure 1 and Table 1 depict the mean propObs for the positive and negative cue words in both BDI groups. There was no significant main group effect for propObs, *F*(1, 104)=0.56, *p*=.45, *d*=.14, and no main valence effect, *F*(1, 104)=2.35, *p*=.13, *d*=.14. However, the analysis did reveal a significant interaction between BDI-II group and cue valence, *F*(1, 104)=3.93, *p*=.05. To analyse valence effects within groups, contrast analyses were conducted. As predicted, people in the high-BDI group retrieved significantly more observer-perspective memories in response to positive cue words than to negative cue words, *F*(1, 104)=5.85, *p*=.02, *d*=.34.^3^^,^ 4 For the low-BDI group, no such difference was found, *F*(1, 104)=0.11, *p*=.74, *d*=−.04. Between groups, contrast analyses revealed no significant observer-perspective difference for memories in response to negative cues, *F*(1, 104)=0.16, *p*=.69, *d*=−.08. For memories in response to positive cues, the difference was in the predicted direction but not significant, *F*(1, 104)=2.66, *p*=.11, *d*=.32.
^3^This valence difference can be characteristic of depressed individuals or it can be a trend that gradually emerges with increasing depressive symptomatology in a non-clinical sample. To verify this, the BDI-II scores were divided into five groups of comparable size. PropObs-pos and propObs-neg were plotted for each of these five BDI-II groups. The figure demonstrated that the relationship between positive and negative memories was reversed only in the group with the highest BDI-II scores. It emphasises that a pattern with more observer perspective for positive than for negative memories only emerges from a certain cut-off, namely in a group with clinically significant levels of depressive symptomatology. Additionally, visual inspection of the graph suggested that this valence difference may not only be driven by changes in perspective for positive memories, but also by a decrease in observer perspective for negative memories.

^4^Memory specificity is frequently investigated as a characteristic of autobiographical memories. Like observer perspective, overgeneral memory is related to depression (Williams et al. 2007). This means that depressed individuals have difficulty recalling specific memories (i.e., memories that refer to events that occurred at a particular time and place and lasted less than one day). Bergouignan et al. (2008) found an overgeneral autobiographical memory bias only for positive events as well as an enhanced use of observer perspective in euthymic patients compared to controls. Consequently, they proposed that the overgeneral bias might be due to an impairment in field perspective for positive memories. Although Lemogne et al. (2006) found that specificity and perspective were not significantly correlated, Lemogne et al. (2009) found significant associations with specificity for negative (not for positive) memories. To check whether the enhanced observer perspective in our study goes together with reduced memory specificity, specificity of each memory was coded. Analyses were twofold: (1) we did not find that positive memories of dysphorics were less specific (compared to the low-BDI group and to negative memories, *p*s>.41); (2) there were no significant correlations between the proportion of specific memories and the proportion of observer perspective for positive or for negative memories, for low-BDI or for high-BDI group (*r*s<.22, *p*s>.11). It seems that the enhanced use of an observer perspective for positive memories did not cause a specificity deficit or vice versa.


### Observer perspective in positive memories and dampening positive affect

Descriptives for dampening scores are presented in Table 1. In the total sample, we found no association between the proportion of observer-perspective memories to positive cues and a dampening response style to positive affect, *r*(421)=.02, *p*=.70 (10 RPA dampening scores missing). However, in the high-BDI group the variables were marginally significantly associated, *r*(48)=.28, *p*=.052 (two RPA dampening scores missing), while this was not the case in the low-BDI group, *r*(54)=−.10, *p*=.49 (two RPA dampening scores missing). The correlations showed a trend to differ, *z*=1.89, *p*=.06 (Fisher's Z-test). No significant correlations were found for negatively cued memories.

## DISCUSSION

The present study investigated the relationship between vantage perspective and depressive symptoms in a student sample. Consistent with the first main hypothesis, dysphoric students were more likely to recall their positive memories from an observer perspective than their negative memories, similar to the study of Lemogne et al. (2006) with diagnosed patients. This pattern was not found in non-dysphoric students. The correlational results appear to support the emphasis on a positive-memory perspective deficit as put forward by Lemogne et al. (2006; see also Bergouignan et al., 2008). However, regarding positive memories, the group difference was not replicated. The valence difference in dysphoric participants appears to be not only driven by the positive, but also by the negative memories.

How can we explain that an observer perspective was used more frequently for positive memories compared with negative memories only in the dysphoric group? An observer perspective allows us to be distanced from the imagined memory. As suggested by Libby and Eibach (2002) field perspective is related to memories according to the current self, while an observer perspective is related to memories that conflict with the current self (see also Bergouignan et al., 2008; Kuyken & Howell, 2006; Lemogne et al., 2006). The working self-concept in depression is mostly negative (e.g., Beck et al., 1979) and depression entails a discrepancy between the current negative self-concept and a more positive ideal self (Carver, Lawrence, & Scheier, 1999; Strauman & Higgins, 1987). Because positive memories do not currently meet their self-concept, depressed individuals might be psychologically distanced from their past selves during positive life times and be more likely to recall positive memories from an observer perspective when compared to negative memories. Negative events, on the other hand, do not conflict with their current self-concept. However this would not be the case for non-depressed individuals. The tentative correlational data (between depressive symptoms and the use of an observer perspective) suggest that the valence difference is more likely to be explained by an increased use of an observer perspective in dysphoria for *positive* (but not negative) memories. However, this was not expressed in the group differences—therefore, we cannot firmly conclude that the valence difference was only driven by positive memories, as there also seems to be an influence of a decrease in observer perspective for negative memories. Future research is needed to further examine the importance of a potential decrease of observer perspective for negative memories, besides the role of positive memories.

Interestingly, the increased use of an observer perspective in positive memories might strengthen the perception that one is not “that positive person” anymore. Such judgements of self-change emerge especially when people focus on differences between their current and recalled “observed” selves (Libby, Eibach, & Gilovich, 2005), which might be the case with depression due to the discrepancy between the current negative self-concept and a more positive ideal self (Carver et al., 1999; Strauman & Higgins, 1987).

Another research tradition fits with this reasoning and gives additional clarification. An observer perspective requires less information to form than a field perspective (Rubin, Burt, & Fifield, 2003). Accordingly, Rubin, Berntsen, and Johansen (2008) propose that the few peripheral details of a tunnel memory might lead to out-of-body experiences. Paralleling this reasoning, given that positive memories fit less with the current self-concept, it might be more difficult to imagine event details compared to the more recognisable negative memories. Therefore, fewer details might also have contributed to more observer memories in the current study.

In accord with the second hypothesis, our correlational data suggest, for dysphoric people, a link between an observer perspective in positive memories and a particular cognitive response style that can dampen the positivity of an experience and/or affect (Feldman et al., 2008; Raes et al., 2009; Raes et al., 2012). It might be that a “distancing” observer perspective facilitates dampening responses to positive affect and/or that a dampening response style promotes the use of an observer perspective in which the self is central. It underlines the evaluative character of the observer perspective. Such an association between observer perspective and dampening is not irreconcilable with the idea that visual perspective is linked with the match between current and recalled self.

It may be interesting to consider the use of imagery-perspective manipulation in therapy, particularly for positive memories in depression. First, perspective change may influence the judgement of the past self and help to create a more coherent self. Second, depressed individuals may benefit from imagining positive events from a field perspective in improving positive affect. In fact, there is some preliminary evidence that a computerised intervention that trains depressed individuals to generate positive imagery from a field perspective can significantly improve symptoms of depression (Blackwell & Holmes, 2010; Lang, Blackwell, Harmer, Davison, & Holmes, 2012).

Our study has several limitations. For example, research has shown that older memories are linked to an observer perspective and recent memories linked to a field perspective (e.g., Berntsen & Rubin, 2006). Participants were only instructed to retrieve memories that were not recent (at least older than one week). Due to the depressive symptomatology, individuals in the high-BDI group may have experienced fewer positive events recently, resulting in older positive than negative memories. Future research could include an assessment of the age of memories to exclude this alternative explanation.

Further, in line with other studies (e.g., Kuyken & Howell, 2006; Kuyken & Moulds, 2009), we categorised the valence of a memory using the cue word, while other authors specifically asked participants to retrieve positive or negative personal events (e.g., Lemogne et al., 2006). Our data and other studies (Dalgleish & Yiend, 2006; Kuyken & Howell, 2006) indicate that memories recalled in response to positive and negative cue words are mostly rated as pleasant and unpleasant respectively. Nevertheless, we suggest that future studies include an assessment of the emotionality of the memories that are rated by the participant.

To conclude, comparing dysphoric with non-dysphoric individuals, it was found that those with dysphoria recalled more of their positive memories from an observer perspective than their negative memories, while this was not the case for the non-dysphoric students. Also, we found preliminary evidence that a dampening style to positive affect may explain in part this pattern in dysphoria.

## References

[CIT0001] American Psychiatric Association 1994 *Diagnostic and statistical manual of mental disorders * 4th ed.; DSM-IV WashingtonDC : APA .

[CIT0002] Bargh J. A. , & Tota M. E. 1988 Context-dependent automatic processing in depression: Accessibility of negative constructs with regard to self but not others *Journal of Personality and Social Psychology* , 54 , 925 – 939 10.1037//0022-3514.54.6.925 3397867

[CIT0003] Beck A. T., Rush A. J., Shaw B. F., Emery G. (1979). *Cognitive therapy of depression*.

[CIT0004] Beck A. T., Steer R. A., Brown G. K. (1996). *Beck Depression Inventory*.

[CIT0005] Bergouignan L. , Lemogne C. , Foucher A. , Longin E. , Vistoli D. , Allilaire J. F. , & Fossati P. 2008 Field perspective deficit for positive memories characterizes autobiographical memory in euthymic depressed patients *Behaviour Research and Therapy* , 46 , 322 – 333 10.1016/j.brat.2007.12.007 18243159

[CIT0006] Berntsen D. , & Rubin D. C. 2006 Emotion and vantage point in autobiographical memory *Cognition and Emotion* , 20 , 1193 – 1215 10.1080/02699930500371190

[CIT0007] Blackwell S. E. , & Holmes E. A. 2010 Modifying interpretation and imagination in clinical depression: A single case series using cognitive bias modification *Applied Cognitive Psychology* , 24 , 338 – 350 10.1002/acp.1680

[CIT0008] Carver S. C. , Lawrence J. W. , & Scheier M. F. 1999 Self-discrepancies and affect: Incorporating the role of feared selves *Personality and Social Psychology Bulletin* , 25 , 783 – 792 10.1177/0146167299025007002

[CIT0009] Cohen J. 1960 A coefficient of agreement for nominal scales *Educational and Psychological Measurement* , 20 , 37 – 46 10.1177/001316446002000104

[CIT0010] Dalgleish T. , & Yiend J. 2006 The effects of suppressing a negative autobiographical memory on concurrent intrusions and subsequent autobiographical recall in dysphoria *Journal of Abnormal Psychology* , 115 , 467 – 473 10.1037/0021-843X.115.3.467 16866587

[CIT0011] Debeer E. , Hermans D. , & Raes F. 2009 Associations between components of rumination and autobiographical memory specificity as measured by a Minimal Instructions Autobiographical Memory Test *Memory* , 17 , 892 – 903 10.1080/09658210903376243 19882439

[CIT0012] Feldman G. C. , Joormann J. , & Johnson S. L. 2008 Responses to positive affect: A self-report measure of rumination and dampening *Cognitive Therapy and Research* , 32 , 507 – 525 10.1007/s10608-006-9083-0 20360998 PMC2847784

[CIT0013] Holmes E. A. , Coughtrey A. E. , & Connor A. 2008 Looking at or through rose-tinted glasses? Imagery perspective and positive mood *Emotion* , 8 , 875 – 879 10.1037/a0013617 19102599

[CIT0014] Holmes E. A. , Lang T. J. , & Shah D. M. 2009 Developing interpretation bias modification as a “cognitive vaccine” for depressed mood: Imagining positive events makes you feel better than thinking about them verbally *Journal of Abnormal Psychology* , 118 , 76 – 88 10.1037/a0012590 19222316

[CIT0015] Kuyken W. , & Howell R. 2006 Facets of autobiographical memory in adolescents with major depressive disorder and never-depressed controls *Cognition and Emotion* , 20 , 466 – 487 10.1080/02699930500342639 26529216

[CIT0016] Kuyken W. , & Moulds M. L. 2009 Remembering as an observer: How is autobiographical memory retrieval vantage perspective linked to depression? *Memory* , 17 , 624 – 634 10.1080/09658210902984526 19536690

[CIT0017] Lang T. J. , Blackwell S. E. , Harmer C. J. , Davison P. , & Holmes E. A. 2012 Cognitive bias modification using mental imagery for depression: Developing a novel computerized intervention to change negative thinking styles *European Journal of Personality* , 26 , 145 – 157 10.1002/per.855 23316101 PMC3532611

[CIT0018] Lemogne C. , Bergouignan L. , Piolino P. , Jouvent R. , Allilaire J.-F. , & Fossati P. 2009 Cognitive avoidance of intrusive memories and autobiographical memory: Specificity, autonoetic consciousness, and self-perspective *Memory* , 17 , 1 – 7 10.1080/09658210802438466 18979356

[CIT0019] Lemogne C. , Piolino P. , Friszer S. , Claret A. , Girault N. , Jouvent R. , Allilaire J.-F. , Fossati P. 2006 Episodic autobiographical memory in depression: Specificity, autonoetic consciousness, and self-perspective *Consciousness and Cognition* , 15 , 258 – 268 10.1016/j.concog.2005.07.005 16154765

[CIT0020] Libby L. K. , & Eibach R. P. 2002 Looking back in time: Self-concept change affects visual perspective in autobiographical memory *Journal of Personality and Social Psychology* , 82 , 167 – 179 10.1037//0022-3514.82.2.167 11831407

[CIT0021] Libby L. K. , Eibach R. P. , & Gilovich T. 2005 Here's looking at me: The effect of memory perspective on assessments of personal change *Journal of Personality and Social Psychology* , 88 , 50 – 62 10.1037/0022-3514.88.1.50 15631574

[CIT0022] Libby L. K. , Shaeffer E. M. , & Eibach R. P. 2009 Seeing meaning in action: A bidirectional link between visual perspective and action identification level *Journal of Experimental Psychology: General* , 138 , 503 – 516 10.1037/a0016795 19883133

[CIT0023] Morse S. , & Gergen K. J. 1970 Social comparison, self-consistency, and the concept of self *Journal of Personality and Social Psychology* , 16 , 148 – 156 10.1037/h00298623 5485940

[CIT0024] McIsaac H. K. , & Eich E. 2002 Vantage point in episodic memory *Psychonomic Bulletin & Review* , 9 , 146 – 150 10.3758/BF03196271 12026947

[CIT0025] McIsaac H. K. , & Eich E. 2004 Vantage point in traumatic memory *Psychological Science* , 15 , 248 – 253 10.1111/j.0956-7976.2004.00660.x 15043642

[CIT0026] Nigro G. , & Neisser U. 1983 Point of view in personal memories *Cognitive Psychology* , 15 , 467 – 482 10.1016/0010-0285(83)90016-6

[CIT0027] Raes F., Daems K., Feldman G. C., Johnson S. L., Van Gucht D. (2009). A psychometric evaluation of the Dutch version of the Responses to Positive Affect questionnaire. *Psychologica Belgica*.

[CIT0028] Raes F. , Smets J. , Nelis S. , & Schoofs H. 2012 Dampening of positive affect prospectively predicts depressive symptoms in non-clinical samples *Cognition and Emotion* , 26 , 75 – 82 10.1080/02699931.2011.555474 21756217

[CIT0029] Robinson J. A. , & Swanson K. L. 1993 Field and observer modes of remembering *Memory* , 1 , 169 – 184 10.1080/09658219308258230 7584265

[CIT0030] Rubin D. C. , Berntsen D. , & Johansen M. K. 2008 A memory-based model of posttraumatic stress disorder: Evaluating basic assumptions underlying the PTSD diagnosis *Psychological Review* , 115 , 985 – 1011 10.1037/a0013397 18954211 PMC2762652

[CIT0031] Rubin D. C. , Burt C. D. B. , & Fifield S. J. 2003 Experimental manipulations of the phenomenology of memory *Memory & Cognition* , 31 , 877 – 886 10.3758/BF03196442 14651296

[CIT0032] Strauman T. J. , & Higgins E. T. 1987 Automatic activation of self-discrepancies and emotional syndromes: When cognitive structures influence affect *Journal of Personality and Social Psychology* , 53 , 1004 – 1014 10.1037/0022-3514.53.6.1004 3694448

[CIT0033] Van der Does A. J. W. (2002). *Handleiding bij de Nederlandse bewerking van de BDI-II *[Manual of the Dutch version of the BDI-II]**.

[CIT0037] Williams J. M. G. , Barnhofer T. , Crane C. , Hermans D. , Raes F. , Watkins E. , & Dalgleish T. 2007 Autobiographical Memory Specificity and Emotional Disorder *Psychological Bulletin* , 133 , 122 – 148 10.1037/0033-2909.133.1.122 17201573 PMC2834574

[CIT0034] Williams J. M. G. , & Broadbent K. 1986 Autobiographical memory in suicide attempters *Journal of Abnormal Psychology* , 95 , 144 – 149 10.1037/0021-843X.95.2.144 3711438

[CIT0036] Williams A. D. , & Moulds M. L. 2008 Manipulating recall vantage perspective of intrusive memories in dysphoria *Memory* , 16 , 742 – 750 10.1080/09658210802290453 18720223

